# ABCG2 Genetic Variability in Drug Exposure and Toxicity: Implications for Clinical Practice

**DOI:** 10.3390/toxics14040327

**Published:** 2026-04-15

**Authors:** Tamara Božina, Livija Šimičević, Lana Ganoci, Mila Lovrić, Iva Klarica Domjanović, Vladimir Trkulja, Nada Božina

**Affiliations:** 1 Department for Chemistry and Biochemistry, University of Zagreb School of Medicine, Šalata 3, 10000 Zagreb, Croatia; tamara.bozina@mef.hr (T.B.); livija.simicevic@kbc-zagreb.hr (L.Š.);; 2 Division for Pharmacogenomics and Therapy Individualization, Department of Laboratory Diagnostics, University Hospital Centre Zagreb, Kišpatićeva 12, 10000 Zagreb, Croatia; 3 Department of Basic and Clinical Pharmacology, University of Zagreb School of Medicine, Šalata 3, 10000 Zagreb, Croatia; vladimir.trkulja@mef.hr; 4 Division for Laboratory Pharmacology and Toxicology, Department of Laboratory Diagnostics, University Hospital Centre Zagreb, Kišpatićeva 12, 10000 Zagreb, Croatia; 5 Croatian Agency for Medicinal Products and Medical Devices, Ksaverska Cesta 4, 10000 Zagreb, Croatia

**Keywords:** ATP-binding cassette transporters, ABCG2 protein, pharmacogenetics, drug-related side effects and adverse reactions, drug interactions, drug transporters, precision medicine, polygenic risk score

## Abstract

The ATP-binding cassette subfamily G member 2 (ABCG2), also known as breast cancer resistance protein (BCRP), is an efflux transporter expressed in key pharmacokinetic tissues and biological barriers. It regulates exposure to many endogenous compounds, drugs, and environmental toxins. Genetic variability in *ABCG2* has been recognised as an important contributor to interindividual variability in drug response, especially in terms of efficacy and toxicity. This narrative review summarises current knowledge on the clinical relevance of *ABCG2* genetic variants, with a focus on their effects on pharmacokinetics, adverse drug reactions and drug–drug–gene interactions, as well as their potential implementation in personalised therapy. A literature search was performed in PubMed, Scopus and the Clinical Pharmacogenomics Database (ClinPGx), with an emphasis on clinically relevant studies and available pharmacogenomic guidelines. The most investigated *ABCG2* variant, c.421C>A (rs2231142; p.Gln141Lys), is consistently associated with reduced transporter activity and increased systemic exposure to several substrate drugs, including statins, allopurinol and anticancer agents, which may influence both treatment response and the risk of toxicity. Although growing evidence supports the clinical relevance of *ABCG2* genotyping, its routine implementation remains limited. Integration of *ABCG2* variability into polygenic models and clinical decision-support tools may further improve individualised treatment, particularly in patients with multimorbidity and polypharmacy.

## 1. Introduction

The superfamily of membrane-bound ATP-binding cassette (ABC) transporters plays a significant role in controlling the transport of a wide range of compounds across cellular membranes, ranging from drugs to environmental contaminants as well as endogenous molecules [1,2]. In humans, forty-eight ABC transporters have been identified and classified into seven subfamilies, ABCA to ABCG [3,4,5]. Based on structural and functional data, a new classification of ABC transporters has been proposed [6]. Among them, ABC subfamily G isoform 2 (ABCG2), also known as breast cancer resistance protein (BCRP), is of clinical relevance due to its role in the cellular efflux of multiple structurally different compounds, which implicates it in multidrug resistance (MDR), as well as in drug efficacy and toxicity. First identified in 1998 in multidrug-resistant breast cancer cell lines, it was originally named BCRP [7,8]. Subsequently, it was linked to MDR in other malignant cell types and came into focus in targeted therapy research [9,10,11]. Coincidentally, it was also identified in the placenta [12] and in a mitoxantrone-resistant colon carcinoma cell line, where it was named the mitoxantrone resistance (MXR) gene [8]. However, it soon became evident that BCRP is widely distributed throughout the body. Due to its similarity to the ABCG family of proteins [13], it was renamed from BCRP to *ABCG2* by the Human Genome Nomenclature Committee (HGNC).

**Figure 1:**
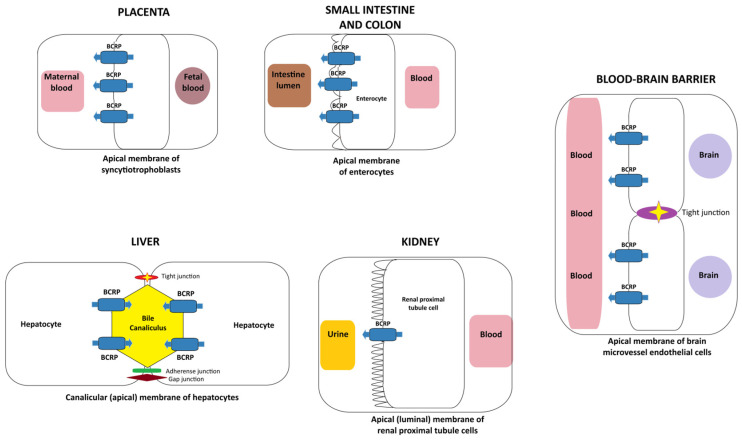
Barrier-oriented distribution of the BCRP (ABCG2) efflux transporter in humans. BCRP (ABCG2) is localised to the apical/luminal membranes of major barrier tissues (placenta, intestine, liver canaliculi, kidney proximal tubule, and blood–brain barrier), where it limits drug absorption, promotes biliary and renal excretion, and restricts foetal and brain exposure.

In polarised epithelial and endothelial cells, ABCG2 is localised to the apical plasma membrane. Acting as an efflux transporter, it extrudes substrates from the intracellular to the extracellular space. It is expressed in many tissues and physiological barriers [14,15]: enterocytes of the small intestine [16,17,18], liver canalicular membranes, bile ducts, and gallbladder [15,19], renal proximal tubular cells [20,21], brain capillary endothelial cells in the blood–brain barrier [22,23,24,25,26], and placental syncytiotrophoblasts [2,12,27] (Figure 1). It is also expressed in mammary alveolar epithelial cells (part of the blood–milk barrier) [28,29], adrenal glands, prostate, testes, ovaries, uterus, venous endothelium, and retinal capillary endothelial cells [30,31,32]. ABCG2 is expressed in various types of stem cells, including haematopoietic stem cells [33,34,35], pluripotent stem cells [4,36], and cancer stem cells [37,38,39]. It is also present in the membranes of red blood cells [15,40,41].

Depending on its location, ABCG2 plays an important role in restricting entry or facilitating the excretion of various substrates, thus acting as a key modulator of systemic exposure to various xenobiotics, including environmental toxicants and drugs. Since it is expressed in pharmacokinetically relevant tissues, this transporter contributes to the absorption, distribution, and elimination of drugs [30]. For example, at the blood–brain barrier, ABCG2 modulates the availability of compounds in the central nervous system, which may contribute to resistance to antiepileptic and psychotropic drugs [42,43,44], whereas in the placenta, it protects the foetus from maternally derived toxins [45,46].

### 1.1. Physiological and Pathophysiological Factors Affecting ABCG2 Expression and Activity

ABCG2 is encoded by the *ABCG2* gene located on chromosome 4q22 and spans around 141 kb of the genomic region, consisting of 16 exons and 15 introns [47,48]. The gene encodes a 72 kDa protein consisting of 655 amino acids [49]. Kukal [22] comprehensively reviewed the physiological roles of the ABCG2 transporter and various factors, signalling pathways, and molecular mechanisms that modulate its levels and activity. These include transcription factors and regulatory proteins (e.g., caveolin-1, insulin-like growth factor mRNA-binding protein 3 (IMP3), and postsynaptic density 95/disc large/zona occludens (PDZ) domain-containing 1 (PDZK1)). In addition, several signalling pathways are involved, including kinase-mediated pathways, Wnt/β-catenin, and SHH/GLI, as well as inflammatory mediators, growth factors, hormones, miRNAs, and epigenetic factors. Regulation of the ABCG2 transporter by lysosomal and proteasomal degradation is also relevant [47,50,51,52,53]. Clinical conditions known to be associated with altered expression and function of ABCG2 include inflammatory and infectious diseases [54,55,56,57,58,59,60,61,62,63,64,65,66], tissue damage [67,68,69,70], cardiomyopathies and myocardial infarction [71,72,73,74], ischemic stroke [75,76], Alzheimer’s disease and amyotrophic lateral sclerosis [77,78,79,80,81,82], hyperuricemia [83], cancer [84,85,86,87,88,89,90], and exposure to endobiotics and xenobiotics [91,92,93,94,95,96,97,98,99,100,101,102]. These changes may contribute to further deterioration of pathophysiological conditions and, in cases of the administration of ABCG2 drug substrates, may result in either drug resistance or increased drug toxicity [9,48].

### 1.2. Genetic Variants of ABCG2

ABCG2 activity is strongly genetically determined. Gene variants can affect protein expression and cellular trafficking. The activity of ABCG2 can be affected either by limited ATPase activity or by altered substrate affinity and/or substrate profile [103,104,105]. A large number of mutations and polymorphisms/gene variants have been described (over 1000), including synonymous mutations and genetic variants in non-coding regions. Although numerous variants have been described, only a limited number have been functionally and clinically well-characterised.

From a practical point of view in clinical pharmacotherapy, nonsynonymous single-nucleotide polymorphisms (SNPs) in the coding region are most important for a personalised approach. A proposed categorisation of *ABCG2* gene variants and a summary of the main features of representative mutations have been published [103]. Class 0 represents the wild-type gene, class 1 includes variants resulting in no protein, class 2 includes variants with a trafficking defect, class 3 includes variants with reduced transport activity, class 4 includes variants with altered substrate recognition, class 5 includes variants with diminished protein expression, class 6 includes variants with a shorter plasma membrane half-life (SNPs result in less stable protein on the cell surface), class 7 includes variants with no RNA, and others include ambiguous and gain-of-function variants. In the pharmacogenetic context, the most extensively studied *ABCG2* variants are c.421C>A (rs2231142, NM_004827.3:c.421C>A, p.Gln141Lys) and c.34G>A (rs2231137; NM_004827.3:c.34G>A, p.Val12Met). The glutamine-to-lysine substitution at position 141 (Q141K) induces protein instability in the endoplasmic reticulum, resulting in enhanced ubiquitin-mediated proteasomal degradation. The global prevalence of this variant is 0.119. The highest prevalence is in Asians (0.22–0.32), Europeans (0.11–0.14), and Africans (~2%) [52,106,107,108,109]. In addition to the well-characterised and common c.421C>A variant, *ABCG2* has more than 500 rare (<1% minor allele frequency) nonsynonymous variants, but their functional consequences remain largely unknown [103]. The Q141K polymorphism [110] and several other mutations/polymorphisms, such as Q126X (rs72552713, NM_004827.3:c.376C>T, p.Gln126Ter), R147W (rs372192400, NM_004827.3:c.439C>T, p.Arg147Trp), T153M (rs753759474, NM_004827.3:c.458C>T, p.Thr153Met), and D620N (rs34783571, NM_004827.3:c.1858G>A, p.Asp620Asn) [111,112,113,114,115], have been identified as risk factors for gout, but this topic is beyond the focus of the present review.

### 1.3. Pharmacological Functions of ABCG2

ABCG2 is involved in the pharmacokinetics of a number of drug substrates, and its function is likely to be reflected in therapeutic efficacy and the risk of adverse drug reactions (ADRs) [104,116,117,118,119,120,121,122,123,124,125,126,127,128,129,130,131,132,133,134,135,136,137,138,139,140,141,142,143,144,145,146,147,148,149]. ABCG2 substrates are drugs from different therapeutic classes, including anticancer drugs [3,150,151,152,153], antibiotics, antiretrovirals [154,155], antiepileptics [156,157,158,159], calcium channel blockers [160], sulfasalazine [161], hypolipidemics [162], proton pump inhibitors (PPIs, e.g., pantoprazole), anticoagulants (apixaban and rivaroxaban), and cyclosporine [32,104,149,163]. Various natural health products have also been identified as ABCG2 substrates [164,165,166]. Co-administration of these orally administered drugs with ABCG2 inhibitors affects their pharmacokinetics. The best-known drugs that are ABCG2 inhibitors include a number of tyrosine kinase inhibitors (TKIs), human immunodeficiency virus protease inhibitors, hepatitis C virus protease inhibitors, calcium channel blockers, antifungal azoles, immunosuppressants, tamoxifen, reserpine, omeprazole, pantoprazole [32,167,168,169,170], and febuxostat [171]. Some natural products [172,173] and food additives may also interact with intestinal ABCG2.

Based on clinical evidence of ABCG2-mediated drug–drug interactions (DDIs), both the US Food and Drug Administration (FDA) and European Medicines Agency (EMA) recommend preclinical evaluation and, when appropriate, clinical assessment of new therapeutics regarding their potential to be involved in ABCG2-mediated DDIs [174,175].

ABCG2-mediated efflux of drugs is recognised as a factor that significantly contributes to poor bioavailability and resistance in tumour and other cells [32]. Inhibition of the ABCG2 transporter is increasingly considered as a possible strategy to overcome multidrug resistance (MDR) [176,177]. The goal is to restore sensitivity of ABCG2-overexpressing cells to anticancer drugs, effectively reversing the MDR phenotype. One example is the use of N-methylpyrazole derivatives as promising selective inhibitors of ABCG2 to restore sensitivity to the anticancer drug SN38 (active metabolite of irinotecan) [178]. The molecular role of BCRP-mediated breast cancer MDR and its inhibitors reported in recent years has been reviewed in detail [9].

Emerging pharmacogenomic (PGx) data suggest that genetic polymorphisms in ABCG2 influence drug exposure and toxicity and may also modulate drug–drug interactions (DDIs), reinforcing the need for personalised treatment approaches based on the *ABCG2* genotype-defined phenotype [30,179,180,181,182,183,184,185,186].

In this narrative review, we summarise current research progress on the role of *ABCG2* gene variants as important biomarkers of drug exposure, efficacy, and toxicity. We present and discuss the most important evidence-based data and recommendations issued by professional societies regarding particular *ABCG2* variants and the respective drugs that are the most likely candidates for translation into clinical practice of personalised pharmacotherapy.

## 2. Materials and Methods

Several national or international professional associations, research networks and expert groups focus on pharmacogenetics/genomics and its implementation in clinical practice, e.g., the Clinical Pharmacogenetics Implementation Consortium (CPIC), the Canadian Pharmacogenomics Network for Drug Safety (CPNDS), the Dutch Pharmacogenetic Working Group (DPWG), and the French National Network of Pharmacogenetics (RNPGx). These organisations systematically evaluate emerging literature and issue guidelines related to specific drugs and pharmacogenetic variants with recommendations regarding dosing regimens, follow-up, alternative treatment options, and the need for genotyping in order to improve clinical outcomes in terms of both efficacy and safety/tolerability.

ClinPGx [187] is a comprehensive PGx resource funded by the National Institutes of Health USA, dedicated to the advancement of PGx clinical implementation. Within ClinPGx, a network of experts continuously identifies (by searching bibliographic electronic databases), reviews, and assesses published primary studies, professional guidelines (issued by, e.g., CPIC, CPNDA, DPWG, RNPGx), and approved drug labels pertaining to the relationship between specific pharmacogenetic variants and drugs. Data are curated, catalogued, integrated and disseminated in the form of variant annotations and summary annotations [187,188,189].

Variant annotations comprise curated primary studies—genome-wide association studies (GWASs) or non-GWASs—as well as systematic reviews and meta-analyses relating specific genetic variants or phenotypes to drug-related outcomes, either reporting an association or no association [187,188]. Each study is assigned a score based on several criteria [187,190]. For example: (i) clinical outcomes like efficacy, toxicity or dosage are weighted more than pharmacokinetic or pharmacodynamic outcomes; (ii) larger sample sizes (cohorts) are given greater weight; (iii) stronger associations (e.g., higher odds ratios, risk ratios or other measures of the strength of association) and their combinations with more reassuring statistical test results (e.g., lower *p*-values) are prioritised; (iv) replicated GWAS findings are weighted more heavily than non-replicated ones, and the score also depends on the GWAS *p*-values, and so on. The assigned scores for each item are graded in the range from 0 to 1 (highest value for an “association”) or to −1 (highest value for “no association”), with details provided in ClinPGx [187,190].

**Figure 2:**
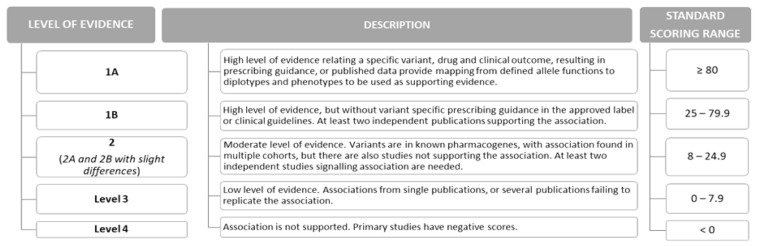
Summary annotation levels of evidence.

Summary annotations are obtained from variant annotations and possible clinical practice guidelines or approved label entries pertinent to a specific variant–drug pair [187,189]. They provide a concise interpretation linking a genetic variant (genotype, allele or phenotype) to a drug, phenotype category, that is, clinical outcome (toxicity, efficacy or pharmacokinetics), and clinical phenotype, that is a specific clinical condition. Each summary annotation is assigned a level of evidence (LOE) for the specific variant–drug–outcome–clinical condition combination. The level of evidence is based on the cumulative score of all relevant variant annotations, with an additional + 100 score points if the variant–drug–outcome combination is addressed in practice guidelines (e.g., by CPIC, DPWG, RNPGx or CPNDS) or in approved drug labels issued by the FDA [187,191,192]. Finally, the LOE is assigned based on the following criteria (in brief; a somewhat different scoring system is implemented for rare genetic variants) as shown in Figure 2 [187,193].

We reviewed the ClinPGx database to identify *ABCG2*-related summary annotations and their LOE, as well as exemplary variant annotations related to level 1A and 2 summary annotations. We also searched PubMed/Medline, Scopus and Google Scholar for the period from 1 January 2015 to 31 December 2025, using keywords that included: “ABCG2/BCRP expression and activity”, “ABCG2/BCRP localization”, “Physiological functions of ABCG2”, “Genetic variants/polymorphism”, “ABCG2 and chemical toxicants”, “ABCG2 drug substrates”. The search aimed to identify original studies, systematic reviews, meta-analyses, and practice guidelines (other than those included in ClinPGx) reporting additional evidence on the relationship between *ABCG2* variants and drug-related clinical outcomes. We also considered reports addressing information not routinely covered in ClinPGx, like reports on the potential involvement of *ABCG2* variants as moderators of drug–drug interactions, i.e., reports illustrating drug–drug–gene interactions involving *ABCG2* variants, and their potential role in polygenic risk scores predictive of drug efficacy and safety.

As this was a narrative review, no formal risk-of-bias assessment was performed. However, emphasis was placed on the consistency of findings across studies, the strength of evidence, and the clinical relevance of reported associations.

## 3. Results

### 3.1. ClinPGx Annotations and Other Observations Related to ABCG2 Variants

As of 26 March 2026, there are 297 ClinPGx variant annotations related to *ABCG2*. The most common drugs addressed in these annotations are methotrexate (34 annotations), rosuvastatin (25), and the tyrosine kinase inhibitors imatinib (25), gefitinib (20) and sunitinib (16), accounting for a total 119 annotations overall.

As shown in Table 1 [194], there are 53 summary annotations related to *ABCG2*, of which nearly half (25 annotations) pertain to the *ABCG2 c.421 C>A* (rs2231142) polymorphism. Carriage of the rs2231142 variant allele results in reduced ABCG2 protein expression and transport activity. Consequently, decreased ABCG2 function, either due to the effect of ABCG2 inhibitors or genetic variants, has been associated with increased systemic exposure to multiple substrate drugs and a higher risk of ADRs [52,53,195,196]. Currently, however, only four summary annotations for *ABCG2* reach the highest LOE (1A), all of them related to rs2231142. These include associations with rosuvastatin toxicity (statin-related myopathy) and pharmacokinetics, as well as allopurinol dosage or efficacy (Table 1). In addition, one LOE 2A annotation links rs2231142 with rosuvastatin efficacy in the treatment of hypercholesterolaemia.

The LOE 1A classification for rs2231142 and rosuvastatin-associated myopathy is based on the total score of 98. A major contribution (100 points) comes from the CPIC dosing guideline [147], which recommends that carriers of reduced function allele(s) should use lower rosuvastatin starting doses (≤20 mg), an alternative statin or combination therapy (e.g., statin + ezetimibe), if a dose >20 mg is needed. Two points were subtracted, since sporadic studies have reported a lack of association between this polymorphism and the risk of myopathy.

Likewise, the LOE 1A annotation for rs2231142 and rosuvastatin pharmacokinetics (total score 115.6) is mainly driven by CPIC guidelines (100 points) [147], with emphasis on systemic exposure. Carriers of the variant allele (A in the C>A or T in the G>T notation, heterozygotes or homozygotes) consistently show around 1.5-fold higher systemic exposure (area under the concentration–time curve) compared with wild-type subjects (CC or GG, depending on the notation).

Taken together, these findings indicate that *ABCG2* rs2231142 is associated with approximately 1.5-fold increased rosuvastatin exposure, which is clinically relevant and contributes to an increased risk of statin-related myopathy. This magnitude of effect is considered clinically relevant and underpins current dosing recommendations. This supports the use of lower starting doses or alternative statin strategies in variant carriers.

The LOE 2A classification for rs2231142 and rosuvastatin efficacy in the treatment of hypercholesterolemia (total score 10.75) is assigned due to four medium-sized cohort studies (180–390 patients) repeatedly reporting greater reductions in low-density lipoprotein cholesterol in variant allele carriers compared to wild-type subjects.

For allopurinol dosing in the treatment of gout, the LOE 1A annotation for rs2231142 (total score 102.5) is based on the DPWG guideline [197] (100 points), which recommends that heterozygous carriers of the variant allele should be treated with a 25% higher dose, i.e., titration schedule of 100, 200, 400 and 500 mg/day, whereas variant homozygous carriers may require up to a 40% higher dose than standard regimens (i.e., a titration schedule of 100, 300, 400, 600 and 700 mg/day); additional evidence (2.5 points) was derived from a study not included in the guidelines. Overall, available evidence indicates that *ABCG2* rs2231142 is associated with altered uric acid transport and increased dose requirements for allopurinol in patients with gout. This effect is reflected in guideline-based dose adjustments in variant carriers. This highlights the importance of considering *ABCG2* genotype in patients with suboptimal urate-lowering response.

Mechanistically, the association between reduced ABCG2 function (due to the c.421C>A variant allele) and increased rosuvastatin exposure, with consequent effects on efficacy and safety, is biologically plausible, as rosuvastatin is a known ABCG2 substrate, and reduced transporter function leads to increased absorption and decreased renal excretion [147].

In contrast, the relation with allopurinol is more complex. Allopurinol is not an ABCG2 substrate, but its active metabolite oxypurinol is. Theoretically, reduced ABCG2 function (variant c.421C>A) could increase oxypurinol exposure due to reduced renal excretion and consequently increase allopurinol efficacy. However, ABCG2 is also important for uric acid active renal secretion, and reduced transporter activity predominantly results in reduced uric acid elimination and consequently the need for higher allopurinol doses in variant carriers in the treatment of gout [197].

Beyond rosuvastatin and allopurinol, the variant *ABCG2 c.421C>A* allele has been associated with increased drug exposure or a higher risk of ADRs for several ABCG2 substrate drugs, including sulfasalazine [198], gefitinib, imatinib, sunitinib [199,200,201], mycophenolic acid [202], methotrexate [203,204], statins beyond rosuvastatin [146,186,205,206,207,208], and oral anticoagulants such as apixaban and rivaroxaban [209,210,211]. Table 1 summarises clinically actionable *ABCG2*–drug associations (LOE 1A–2A), highlighting rosuvastatin and allopurinol as the only current high-priority implementations. Lower-level evidence annotations (LOE 3–4), including notes on potential sources of inconsistency (e.g., small sample sizes, population heterogeneity, and concomitant drug effects), are provided in Supplementary Table S1.

**Figure 3:**
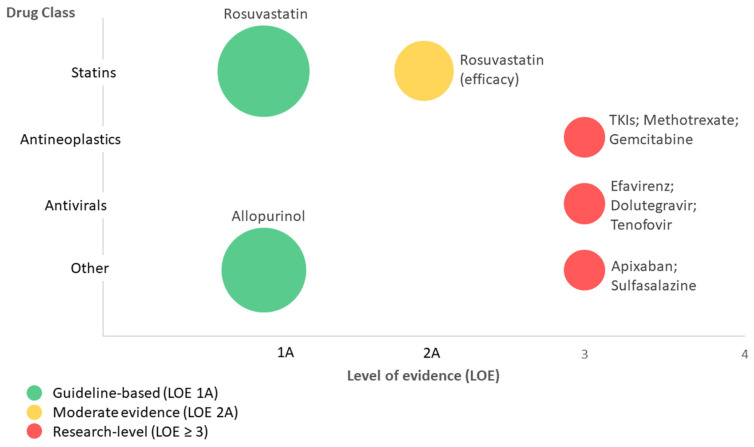
Clinical impact matrix of *ABCG2*–drug associations. Associations are plotted according to the level of evidence (LOE) and drug class. Bubble size reflects the approximate magnitude of effect, while colour indicates clinical actionability (green—guideline-based; amber—moderate evidence; red—research-level). Clinically actionable associations are currently limited to rosuvastatin and allopurinol.

To facilitate a more intuitive overview of the clinical relevance and prioritisation of *ABCG2*–drug associations, a visual summary highlighting clinically actionable versus investigational associations is provided in Figure 3.

Table 2 provides a summary of ClinPGx data on *ABCG2* variants associated with toxicity outcomes of potential clinical relevance, excluding the well-established rs2231142–rosuvastatin myopathy association (LOE 1A), which is already presented in Table 1.

Individual studies have suggested additional associations between *ABCG2* variants and an increased risk of ADRs that are not listed in Table 1 or Table 2. In one study, carriers of the *ABCG2 34C>T* (rs2231137) variant allele (CT or TT genotypes) treated with rosuvastatin had higher rosuvastatin plasma levels, greater lipid-lowering effect and a higher risk of elevated creatine kinase and liver injury [223]. In another study [145], the rs2622629 variant was associated with an increased risk of hepatotoxicity in individuals homozygous for the variant genotype (TT) when treated with statins (atorvastatin, fluvastatin, lovastatin, pitavastatin or simvastatin), compared with CC and CT genotypes. Additionally, the *ABCG2* 34G>A (rs2231137) variant has also been linked to increased susceptibility to liver injury in patients receiving efavirenz [224].

Several studies suggest that rare variants (minor allele frequency < 1%) may modulate ABCG2 expression and function [167,225,226,227]; nevertheless, their clinical consequences are currently unknown.

Both concomitant drug use and genetic factors may contribute to variation in drug efficacy and safety through DDI and drug–gene (DGI) interactions. There is growing evidence that pharmacogenes may act as moderators of DDIs, or, in reverse, that concomitant treatments may act as moderators of DGIs, highlighting the role of DDGI as important determinants of treatment response [228]. Two studies in adult patients with epilepsy indicated a greater effect of valproate on steady-state exposure to lamotrigine in *ABCG2 c.421C>A* (rs2231142) variant allele carriers than in wild-type subjects. Particularly, the valproate-induced increase in lamotrigine dose-corrected trough concentrations was around 5.2-fold in the variant carriers and around 2.3-fold in wild-type subjects [181,183]. These findings further illustrate the potential clinical relevance of *ABCG2* within complex DDGI settings.

### 3.2. Implementation of ABCG2 Pharmacogenomics (PGx) in Clinical Practice

As a concept, pharmacogenetic testing has been confirmed as a valuable clinical tool for the optimisation of medication management, significantly improving drug safety and clinical outcomes, thus contributing to overall healthcare cost reduction [229,230,231]. The PGx approach is particularly valuable in patients with multimorbidity and polypharmacy, who also have higher hospitalisation rates and mortality. It is estimated that 5–15% of hospitalisations in adults and 15% in patients with multimorbidity are due to ADRs [232,233,234]. Furthermore, more than 80% of ADRs associated with hospital admissions or occurring during hospitalisation are dose-dependent and potentially preventable [235,236,237]. Therefore, pharmacogenetic information represents an important element of risk stratification and therapeutic decision-making.

Considerable progress in PGx implementation has been achieved through the establishment of professional societies such as the Clinical Pharmacogenetics Implementation Consortium (CPIC), the Dutch Pharmacogenetics Working Group (DPWG), the Canadian Pharmacogenomics Network for Drug Safety (CPNDS), the French National Network of Pharmacogenetics (RNPGx), and other professional organisations. These societies develop evidence-based guidelines and recommendations (genetic-based dose adjustments) or suggest alternative medications, thereby enabling the translation of knowledge from bench to bedside, facilitating adoption by clinicians and application in practice.

Specifically, with regard to *ABCG2* variants, more than two decades of research have resulted in actionable practical guidance/recommendations regarding the dosing of rosuvastatin in dyslipidaemia and allopurinol in gout with respect to the *ABCG2 c.421C>A* genotype. At the same time, many additional variant–drug associations related to efficacy and safety have been reported but still lack sufficient high-quality evidence to confirm or rebut their clinical relevance and potential for routine implementation.

### 3.3. ABCG2 Variants from Single Gene–Drug Associations to Polygenic Risk Scores

Drug response phenotypes are highly polygenic and result from the combined effects of multiple genetic variants with small to moderate contributions [238]. This could be one of the reasons for discrepancies among PGx studies, especially in genetically diverse populations. Therefore, polygenic models, such as polygenic risk scores (PRS), are increasingly being used instead of single-gene models to predict variability in drug response [239]. In DDGI studies, PRS aggregate genome-wide variants with small-effect-size and may improve the prediction of drug response compared with traditional single-gene pharmacogenetics [240].

Early pharmacogenomic PRS were extrapolated from disease-associated variants identified by GWAS. More recently, there is a noticeable trend towards the use of pharmacogenomics-specific GWAS data [241,242]. However, several challenges remain, including reliance on extrapolations from disease genetics, limited sample sizes and heterogeneity in phenotype definitions. Despite these challenges, significant progress has been made in developing promising approaches for PRS [242]. Further improvement is important and will depend on the standardisation of phenotyping methodologies and a multidisciplinary approach, including improved statistical methods to assess associations between drug-specific gene variants in the construction of PRS [242,243].

Furthermore, the integration of genomic data with reliable clinical predictors, together with the implementation of simple tools for clinical decision-making and patient education, has been emphasised [242,244]. At the same time, patient privacy, informed consent and the safe use of PRS must be addressed within appropriate ethical and regulatory frameworks.

Polygenic models may be particularly clinically valuable in patients with comorbidities and polypharmacy, where PRSs could support stratification of ADRs risk and guide drug selection and dosing. From this perspective, *ABCG2* is a strong candidate for inclusion in PRS models. This is relevant for, for example, cardiovascular drugs, where multiple ABCG2 substrates, including lipid-lowering drugs, anticoagulants, antiplatelet drugs and antihypertensives, are often prescribed concomitantly [245]. Incorporating *ABCG2* into polygenic models may improve prediction of complex DDGIs between multiple drugs and genes and provide additional valuable information.

This approach is increasingly recognised by professional societies, which acknowledge the importance of integrating polygenic data in clinical medicine, both in diagnostics and in personalised treatment. Policy statements underline the need for strategic investments in biobanking and genomic research across different populations to support the development and implementation of PRS [246].

## 4. Discussion

The ultimate goal of PGx research is to identify genetic traits that can guide the selection of pharmacological treatments (active substances, doses, and dosing regimens) most likely to result in favourable outcomes in individual patients, namely therapeutic success with a minimised risk of adverse events. This implies that, to achieve this goal, such information should be available before the start of treatment, preferably through pre-emptive testing [247]. With respect specifically to *ABCG2* variants, the *ABCG2 c.421C>A* polymorphism appears to be a strong candidate for pre-emptive testing in patients who require treatment with rosuvastatin, as supported by the evidence in the CPIC guideline [147]. Dosing recommendations in the rosuvastatin summary of product characteristics acknowledge the need for reduced doses in people carrying the variant allele, but pre-emptive testing is not explicitly recommended [248]. At the same time, concerns have been raised as to whether the recommended lower doses would indeed prove beneficial, encouraging the need for additional confirmation [249]. Consequently, a general recommendation for pre-emptive testing of the rs2231142-rosuvastatin variant–drug pair has not yet been established. One concern regarding pre-emptive testing, even at a reduced scale of variant–drug pairs (as opposed to wider panels), is its cost-effectiveness. In this respect, it is worth noting the ever-declining costs of genotyping procedures [247]. The CPIC guideline on statin-associated musculoskeletal symptoms also supports a high level of evidence for another variant–statin-related myopathy association, specifically the *SLCO1B1 c.521T*>C (rs4149056) polymorphism. *SLCO1B1* encodes the hepatic influx transporter organic anion transporter polypeptide 1B1. The variant allele (C) is present in several *SLCO1B1* star alleles, including the common *5 and *15 alleles, which are associated with reduced or absent transporter function [147]. ClinPGx lists 96 summary annotations for *SLCO1B1* variants, 26 of which have LOE 1A, all pertaining to rs4149056 or *SLCO1B1* star alleles *1, *5, and *15 in relation to the pharmacokinetics and/or musculoskeletal toxicity of all statins, including rosuvastatin [250]. In agreement, the recommendation for reduced statin dosing in subjects homozygous for the *c.521T>C* variant allele is also included in the rosuvastatin prescribing information [248]. A pharmacoeconomic study conducted in the US demonstrated that pre-emptive *SLCO1B1* genotyping in statin-treated patients was cost-effective compared with either no genotyping or reactive genotyping over a 50-year time horizon [251]. By analogy, it may be reasonably assumed that pre-emptive genotyping of rs2231142 in patients to be treated with rosuvastatin is likely to prove cost-effective in most settings.

Greater concerns have been expressed regarding the justification for the DPWG guideline on allopurinol dosing in patients with gout with respect to rs2231142. It has been discussed that the estimated variant–outcome association was confounded by several factors known to affect serum uric acid concentrations but were not accounted for [249,252]. This different level of confidence about the implementability of rs2231142-guided rosuvastatin and allopurinol dosing likely reflects different levels of understanding of their mechanistic backgrounds, i.e., biological plausibility. In the former case, the mechanism is relatively straightforward (variant—reduced function—higher exposure—greater effect/risk of myopathy), whereas in the latter, it is counterintuitive (reduced transporter function would be expected to increase exposure to oxypurinol [197]). With respect specifically to the practical relevance of information about *ABCG2* variants in guiding successful and safe (individual) pharmacotherapy, this discrepancy may also be viewed as an indicator of two somewhat different settings. One scenario refers to relatively simple and mechanistically well understood situations, in which the following are known: (i) the consequences of a particular variant for compound-specific transporter function; (ii) the consequences of a particular variant for compound-specific systemic and/or local tissue/cell availability; (iii) the pharmacodynamic consequences of altered systemic and/or local exposure in terms of both therapeutic and adverse drug effects. Such scenarios are essentially based on a causal variant–outcome relationship. Although causality and prediction are different concepts and reliable prediction systems do not necessarily require explicit evidence of causality between each of their elements and the outcome [253], reliable medical prediction is typically based on biologically plausible predictors [254]. Such information can then be included in prediction systems with addition of other compound-specific PGx information relevant for the target population (e.g., function-changing variants in genes encoding other transporters or enzymes with reasonable ethnicity-specific prevalence), or “classical” factors known to be causal or likely causal to systemic or local drug availability or susceptibility to specific therapeutic or harmful drug effects (i.e., pharmacokinetic/pharmacodynamic interactions), like concomitant treatments, disease characteristics and comorbidity, and demographic/anthropometric features [255]. The full utility of the rs2231142–rosuvastatin safety association is likely to be achieved only when it is considered parallel with other components of a potential, adequately validated prediction system based on additional constituents carefully selected among established actionable PGx information, like that regarding the *SLCO1B1 c.521T*>C (rs4149056) polymorphism, or possibly other *ABCG2* variants known to reduce transporter activity, although their prevalence is generally very low [103], or a broader, albeit still limited, PGx panel including variants relevant for commonly co-administered “cardiovascular drugs” and their effects, as well as clinical/demographic patient characteristics. The ClinPGx summary annotations for *ABCG2* variant–drug pairs assigned LOE 3 (Table 1) are based on individual studies or limited numbers of studies of variable quality. However, some of these associations appear to be good candidates for such a mechanistically straightforward approach, for example, the association between rs2231142 and atorvastatin-related myopathy (Table 1 and Table 2). Another setting involves associations with less clearly understood mechanisms, such as the association of the rs7699188 variant (class 5 *ABCG2* polymorphism [103]) with a higher risk of severe non-haematological adverse effects in patients treated with fluorouracil/irinotecan-based protocols for colorectal carcinoma, or of the rs2231137 variant (class 0 polymorphism [103]) with an increased risk of diarrhoea in irinotecan-treated patients with non-small-cell lung carcinoma (Table 2). Generally, it seems reasonable to conclude that such associations should be considered credible only if confirmed within a broader context of known actionable PGx data related to irinotecan or fluorouracil (fluoropyrimidine) and “classical” clinical/individual factors that have been clearly shown to be predictive of these relatively complex outcomes [256,257]. Outcome (efficacy or safety) prediction scores based on a larger number of genetic variants (that is, polygenic prediction systems or polygenic risk scores) thus far developed for the purpose of advancing individualised pharmacotherapy have used several general approaches [224,255]: (i) inclusion of variants GWAS-associated with the disease; (ii) inclusion of variants GWAS-associated with indicators of exposure to a particular drug substance (i.e., PGx variants); (iii) inclusion of combined variants GWAS-associated with the disease and specific drug exposure; (iv) inclusion of multiple PGx variants for which it is biologically plausible that they affected exposure to a specific drug or there was explicit evidence of a variant effect on systemic or local drug exposure, typically combined with other “classical” factors known to determine drug exposure. This last strategy seems to be the most plausible if the field of pharmacogenetically guided individualised pharmacotherapy is viewed from the perspective of the ABCG2 transporter and current knowledge of the functional consequences of its genetic variants.

Most of the highest-level evidence (LOE 1A) for the *ABCG2* rs2231142 variant comes from studies conducted in European and East Asian populations, where this variant is relatively common (minor allele frequency ~11–32%). In contrast, it is much less frequent in African populations (~2%), which may limit generalisability of these findings.

At the same time, the majority of other *ABCG2*–drug associations are supported by smaller and more heterogeneous studies, often with inconsistent results and varying methodological approaches. Taken together, these considerations highlight the need for further validation in larger, well-designed studies across diverse populations before wider clinical implementation can be fully justified.

## 5. Conclusions

This review highlights the role of ABCG2 efflux transporter as an important factor of systemic and/or local availability of its drug substrates, as well as a mediator of drug–drug interactions resulting in clinically relevant changes in drug exposure. It also highlights that its activity may be markedly affected by numerous genetic variants. As such, *ABCG2* represents a key pharmacogene of interest. At the present moment, despite extensive research, translation of *ABCG2* pharmacogenomics into clinical practice remains limited. Clinically implementable evidence exists for selected drugs, but most variant–drug associations are characterised by inconsistent findings and low levels of evidence. This appears disproportionate to the well-established pharmacokinetic role of ABCG2. Therefore, *ABCG2* and its genetic variants warrant further research, including well-designed studies with clearly defined clinical outcomes, ideally integrating pharmacokinetic and clinical endpoints, and the evaluation of *ABCG2* variants together with broader panels of substance-specific pharmacogenes and “classical” clinical and demographic factors.

## Figures and Tables

**Table 1:** Clinically actionable *ABCG2*–drug associations (LOE 1A–2A) [194].

Level of Evidence	Variant	Drugs	Phenotype Category	Clinical Phenotype/Indication
1A	rs2231142	Rosuvastatin	Toxicity	Statin-related myopathy
1A	rs2231142	Rosuvastatin	Metabolism/PK	Increased exposure
1A	rs2231142	Allopurinol	Dosage	Gout
1A	rs2231142	Allopurinol	Efficacy	Gout
2A	rs2231142	Rosuvastatin	Efficacy	Hypercholesterolemia and myocardial infarction

All annotations are presented individually as retrieved from ClinPGx to preserve granularity. Abbreviations: PK—pharmacokinetics.

**Table 2:** Summary annotations for *ABCG2* variants associated with toxicity phenotypes (excluding rs2231142–rosuvastatin–myopathy, LOE 1A).

Variant	LOE	Drug	Clinical Condition	Summary Annotation	Interpretation	Ref.
rs2231137	3	Irinotecan	NSCLC	CT/TT genotypes associated with increased diarrhoea; no association with neutropenia vs CC.	Increased risk of diarrhoea; no effect on neutropenia	[212]
rs2231142	3	Atorvastatin	Statin-related myopathy	Variant carriers (CA/AA) may have increased toxicity risk vs CC.	Increased risk of toxicity	[147]
rs2231142	3	Cyclophosphamide, doxorubicin, and 5-fluorouracil (FAC)	Breast neoplasms	Variant carriers (CA/AA) may have increased risk of anaemia vs CC.	Increased risk of toxicity (anaemia)	[213]
rs2231142	3	Efavirenz	HIV infection	Variant carriers (CA/AA) may have increased risk of abnormal dreams vs GG.	Increased risk of CNS adverse reactions	[214]
rs2231142	3	Gemcitabine	NSCLC	Variant carriers (CA/AA) show increased PFS and thrombocytopenia.	Increased efficacy and increased toxicity	[215]
rs2231142	3	Methotrexate	Rheumatoid arthritis	GG genotype associated with increased risk of adverse drug reactions.	Increased risk of toxicity	[216]
rs2231142	3	Sunitinib	Neoplasms	TT genotype may have increased toxicity vs. GG.	Increased risk of toxicity	[217,218]
rs2231142	4	Gefitinib	Lung neoplasms	Possible increased diarrhoea risk; inconsistent findings across studies.	Conflicting results	[201]
rs2231142	4	Methotrexate	ALL; Burkitt lymphoma; T-cell lymphoma; osteosarcoma	No consistent association with exposure; conflicting results.	No clear association; conflicting	[219,220,221]
rs7699188	3	Fluorouracil, irinotecan, and leucovorin	Colorectal neoplasms	A allele associated with higher risk of grade 3–4 toxicity vs G allelle.	Increased risk of severe toxicity	[222]

Summary annotations are simplified from ClinPGx; interpretation has been standardised to overall trends across studies. Abbreviations: ALL—acute lymphoblastic leukaemia; CNS—central nervous system; FAC—fluorouracil, doxorubicin, cyclophosphamide; HIV—human immunodeficiency virus; LOE—level of evidence; NSCLC—non-small-cell lung carcinoma; PK—pharmacokinetics; PFS—progression-free survival.

## Data Availability

No new data were created or analysed in this study. Data sharing is not applicable to this article.

## Supplementary Materials

The following supporting information can be downloaded at: https://www.mdpi.com/article/10.3390/toxics14040327/s1, Table S1: ABCG2 ClinPGx annotations with lower levels of evidence (LOE 3–4) and notes on potential sources of inconsistency [194].

## Abbreviations

The following abbreviations are used in this manuscript:

ABC	ATP-binding cassette
ABCG2	ATP-binding cassette subfamily G member 2
ADR	Adverse drug reaction
AUC	Area under the concentration time curve
BCRP	Breast cancer resistance protein
ClinPGx	Clinical Pharmacogenomics Database
CPIC	Clinical Pharmacogenetics Implementation Consortium
CPNDS	Canadian Pharmacogenomics Network for Drug Safety
DDI	Drug–drug interaction
DPWG	Dutch Pharmacogenetics Working Group
GWAS	Genome-wide association study
HIV	Human immunodeficiency virus
LDL	Low-density lipoprotein
LOE	Level of evidence
MDR	Multidrug resistance
PFAS	Per and polyfluoroalkyl substances
PGx	Pharmacogenomics
PK	Pharmacokinetics
PRS	Polygenic risk score
RNPGx	French National Network of Pharmacogenetics
