# Supplementary material for: ABCG2 Genetic Variability in Drug Exposure and Toxicity: Implications for Clinical Practice
Source: Toxics. 2026 Apr 15;14(4):327. doi: 10.3390/toxics14040327 (PMC13120264; doi:10.3390/toxics14040327)
Supplement: Supplementary file 1 [file toxics-14-00327-s001.zip › toxics-4202254-supplementary.pdf]

Supplementary Table S1. *ABCG2* ClinPGx annotations with lower levels of evidence (LOE 3–4) and notes on potential sources of inconsistency [194].

Level of evidence	Variant	Drugs	Phenotype category	Clinical Phenotype/indication	Comment
3	rs1061018	Dasatinib, imatinib, nilotinib	Other	—	Limited replication across studies
3	rs12505410	Imatinib	Efficacy	Chronic myelogenous leukemia, BCR-ABL1 positive	Small sample sizes
3	rs12505410	Methotrexate	Metabolism/PK	Osteosarcoma	Primarily pharmacokinetic evidence
3	rs13120400	Ceftriaxone	Metabolism/PK	Central nervous system infectious disorder	Limited replication across studies
3	rs13120400	Deferasirox	Efficacy, metabolism/PK	Beta-thalassemia and related diseases	Heterogeneous endpoints
3	rs13120400	Imatinib	Efficacy	CML, BCR-ABL1 positive	Small sample sizes
3	rs13120400	Methotrexate	Efficacy	Psoriasis	Conflicting results
3	rs13120400	Methotrexate	Metabolism/PK	Osteosarcoma	Primarily pharmacokinetic evidence
3	rs13137622	Methotrexate	Metabolism/PK	Osteosarcoma	Primarily pharmacokinetic evidence
3	rs17731538	Methotrexate	Efficacy	Psoriasis	Conflicting results
3	rs2199936	Rosuvastatin	Efficacy	—	Conflicting results
3	rs2231135	Methotrexate	Toxicity	Osteosarcoma	Small sample sizes
3	rs2231137	Dasatinib, imatinib, nilotinib	Other	—	Limited replication across studies
3	rs2231137	Imatinib	Dosage	Gastrointestinal stromal tumours	Small sample sizes
3	rs2231137	Irinotecan	Toxicity	Non-small cell lung carcinoma	Small sample sizes
3	rs2231142	Apixaban	Metabolism/PK	Atrial fibrillation	Limited replication across studies
3	rs2231142	Atorvastatin	Other	—	Conflicting results
3	rs2231142	Atorvastatin	Toxicity	—	Conflicting results
3	rs2231142	Cyclophosphamide, doxorubicin, fluorouracil	Toxicity	Breast neoplasms	Small sample sizes
3	rs2231142	Dolutegravir	Metabolism/PK	HIV infectious disease	Primarily pharmacokinetic evidence
3	rs2231142	Efavirenz	Toxicity	HIV infectious disease	Small sample sizes
3	rs2231142	Fluvastatin	Metabolism/PK	—	Primarily pharmacokinetic evidence
3	rs2231142	Gemcitabine	Efficacy, toxicity	Non-small cell lung carcinoma	Mixed endpoints; limited replication across studies
3	rs2231142	Imatinib	Metabolism/PK	—	Limited replication across studies
3	rs2231142	Lamotrigine	Metabolism/PK	Epilepsy	DDGI confounding (valproate interaction)
3	rs2231142	Methotrexate	Toxicity	Rheumatoid arthritis	Conflicting results
3	rs2231142	Opioid anaesthetics, other general anaesthetics, volatile anaesthetics	Efficacy	—	Limited replication across studies

3	rs2231142	Simvastatin	Metabolism/PK	—	Primarily pharmacokinetic evidence
3	rs2231142	Sulfasalazine	Efficacy	Rheumatoid arthritis	Limited replication across studies
3	rs2231142	Sulfasalazine	Metabolism/PK	—	Primarily pharmacokinetic evidence
3	rs2231142	Sunitinib	Toxicity	Neoplasms	Small sample sizes
3	rs2231142	Tenofovir	Metabolism/PK	HIV infectious disease	Limited replication across studies
3	rs2725252	Imatinib	Efficacy	Chronic myelogenous leukemia, BCR-ABL1 positive	Small sample sizes
3	rs3114020	Lamotrigine	Metabolism/PK	Epilepsy	DDGI confounding (valproate interaction)
3	rs41282401	Dasatinib, imatinib, nilotinib	Other	—	Limited replication across studies
3	rs4148155	Allopurinol	Efficacy	—	Conflicting results
3	rs4148157	Allopurinol	Efficacy	—	Conflicting results
3	rs4148157	Topotecan	Metabolism/PK	Brain neoplasms	Limited replication across studies
3	rs45605536	Dasatinib, imatinib	Other	—	Limited replication across studies
3	rs58818712	Dasatinib, imatinib	Other	—	Limited replication across studies
3	rs72552713	Sulfasalazine	Metabolism/PK	—	Primarily pharmacokinetic evidence
3	rs76979899	Allopurinol	Efficacy	—	Limited replication across studies
3	rs7699188	Fluorouracil, irinotecan, leucovorin	Efficacy, toxicity	Colorectal neoplasms	Mixed endpoints; Limited replication across studies
4	rs10011796	Allopurinol	Efficacy	—	Limited replication across studies
4	rs2231142	Capecitabine, fluorouracil, leucovorin, oxaliplatin	Efficacy	Colorectal neoplasms	Conflicting results; heterogeneous populations
4	rs2231142	Gefitinib	Toxicity	Lung neoplasms	Conflicting results
4	rs2231142	Methotrexate	Toxicity	Acute lymphoblastic leukemia; Burkitt Lymphoma; Drug Toxicity; Lymphoma, T-Cell; Osteosarcoma	Conflicting results; heterogeneous endpoints
4	rs2231142	Methotrexate	Metabolism/PK	Acute lymphoblastic leukemia; Burkitt Lymphoma; Lymphoma, T-Cell; Osteosarcoma	PK variability; conflicting results

All annotations are presented individually as retrieved from ClinPGx to preserve granularity. Comments indicate common sources of inconsistency across studies, including small sample sizes, heterogeneous study designs, limited replication across studies, and potential confounding factors such as concomitant therapies. These comments provide a high-level interpretation rather than a study-specific assessment. Abbreviations: ALL – acute lymphocytic leukemia; BCR-ABL1 – breakpoint cluster region–Abelson 1 fusion gene; CML – chronic myeloid leukemia; HIV – human immunodeficiency virus; PK – pharmacokinetics
